# Diagnostic and prognostic assessments of adrenocortical carcinomas by pathological features, immunohistochemical markers and reticular histochemistry staining

**DOI:** 10.1186/s13000-024-01496-z

**Published:** 2024-05-27

**Authors:** Wenting Gan, Xue Han, Yuxi Gong, Yefan Yang, Cong Wang, Zhihong Zhang

**Affiliations:** Department of Pathology, The First Affiliated Hospital of Nanjing Medical University, 300 Guangzhou Road, Nanjing, Jiangsu Province 210029 China

**Keywords:** Adrenocortical carcinoma, Ki-67, CYP11B1, Reticulin

## Abstract

**Background:**

Current diagnostic criteria of adrenocortical neoplasms are mostly based on morphology. The utility of immunohistochemistry (IHC) and histochemistry is limited.

**Materials and methods:**

To evaluate the diagnostic and prognostic utility of clinicopathological features, morphology, ancillary biomarkers, and reticular histochemistry in adrenocortical neoplasms. We examined 28 adrenocortical carcinomas (ACCs) and 50 adrenocortical adenomas (ACAs) obtained from pathology archives. Clinical data were retrieved from medical records. Two pathologists independently assessed hematoxylin and eosin-stained slides, employing modified Weiss criteria for all tumors and Lin-Weiss-Bisceglia criteria for oncocytic variants. Immunohistochemical markers (Calretinin, alpha-inhibin, MelanA, SF-1, Ki-67, PHH3, IGF-2, β-catenin, P53, CYP11B1, CYP11B2, MLH1, MSH2, MSH6, PMS2, EPCAM) and Gomori’s Silver histochemistry were applied. Statistical analysis utilized SPSS Statistics 26.

**Results:**

ACCs exhibited larger tumor sizes (*P*<0.001) and symptomatic presentations (*P* = 0.031) compared to ACAs. Parameters of modified Weiss criteria and angioinvasion demonstrated diagnostic value for ACCs. Six immunohistochemical antibodies((MelanA, Ki-67, IGF-2, β-catenin, P53 and CYP11B1) and reticulin framework alterations showed diagnostic value. Notably, Ki-67 and reticulin staining were most recommended. Evident reticulin staining was frequently present in ACCs (*P*<0.001). Ki-67 was significantly higher in ACCs (*P*<0.001). Twenty-one conventional and seven oncocytic entities showed different necrosis frequencies. Symptoms and Ki-67 index ≥ 30% were prognostic for ACCs, correlating with shorter survival.

**Conclusions:**

This study emphasizes the diagnostic value of reticulin framework alterations and a high Ki-67 index. Markers such as CYP11B1, IGF2, P53, β-catenin and MelanA also contribute to the diagnosis of ACCs. Symptoms and Ki-67 index ≥ 30% predict shorter survival. These findings encourges the use of ancillary markers such as reticulin histochemistry and Ki-67 in the workup of evaluations of adrenocortical neoplasms.

**Supplementary Information:**

The online version contains supplementary material available at 10.1186/s13000-024-01496-z.

## Introduction

Adrenocortical carcinoma is an endocrine entity originated from adrenal cortex with low morbidity and high mortality, characterized by high malignancy, invasiveness and difficulties in early diagnosis [1, 2]. The cornerstone of ACC diagnosis has traditionally relied upon histopathological evaluation, with established criteria including Weiss criteria, modified Weiss criteria, and Lin-Weiss-Bisceglia criteria, predominantly grounded in morphological assessments [3–6]. Crucial features associated with malignancy encompass elevated mitotic counts, typically exceeding five mitotic figures per 50 high-power fields (HPF), atypical mitotic figures, tumorous necrosis, and capsular invasion. Necrosis is deemed to be present when observed in confluent nests of cells [4]. It is critical to note that the accurate identification of capsular invasion necessitates the unequivocal confirmation of the presence of tumor cells within the capsule, as normal adrenal cortical cells may also be encountered in this location. Recent advancements have spotlighted the significance of angioinvasion (vascular invasion) in both the diagnostic and prognostic context of ACCs [2, 3, 6]. While these histopathological criteria have proven effective in the diagnosis of most ACCs, challenges persist, particularly in distinguishing low-grade ACCs from adrenocortical adenomas (ACAs). Furthermore, ACCs exhibit notable heterogeneity, being classified into four distinct subtypes based on morphological characteristics: conventional, oncocytic, myxoid, and sarcomatoid. Oncocytic variants are characterized by specific features discernible through Lin-Weiss-Bisceglia criteria [3, 7].

In recent years, ancillary biomarkers have emerged as valuable adjuncts to the diagnostic armamentarium for ACCs. The initial step in establishing an ACC diagnosis is confirming its adrenocortical origin, which can be achieved through immunohistochemistry staining targeting specific markers, including SF-1, Calretinin, alpha-inhibin, and MelanA [3, 8]. Additionally, immunohistochemical markers such as IGF-2, β-catenin, p53, and Ki67 have demonstrated utility in distinguishing ACCs from ACAs [2, 3]. Functional antibodies, including CYP11B1 and CYP11B2, markers indicative of zona fasciculata-like and glomerulosa-like cells, are also being increasingly utilized [9]. The mitotic marker PHH3 (Phosphohistone H3) is gaining prominence in evaluating cell proliferation in tumors [10]. Moreover, the application of reticulin histochemistry has proven to be a practical diagnostic method, consistently revealing loss of the reticulin framework in ACCs [2, 11].

Recent research has additionally uncovered a subset of ACCs displaying microsatellite instability, which can be initially assessed through IHC staining for mismatch repair (MMR) proteins, including MLH1, MSH2, MSH6, PMS2, and EPCAM [12]. Microsatellite instability has been observed in various tumor types, including colorectal, gastric, endometrial, and ovarian cancers, and is considered a predictor of responsiveness to chemotherapy and immunotherapy [11].

In this comprehensive study, we aim to evaluate the diagnostic value of pathologic morphological features in adrenocortical neoplasms among adult Chinese patients. Furthermore, we investigate the immunobiomarker profile, encompassing markers of adrenocortical origin, IGF-2, β-catenin, p53, ki67, CYP11B1, PHH3, and MMR proteins, and the role of altered reticulin framework in adrenocortical neoplasms. Prognostic implications of morphological, immunohistochemical, and histochemical features will be assessed, and a comparative analysis among ACC subtypes will be conducted.

## Materials and methods

### Study approval and patient selection

This study obtained approval from the Research Ethics Board of the First Affiliated Hospital of Nanjing Medical University (No.2023-SR-314). Patients diagnosed with ACC from 2014 to 2022 and ACA from 2021 to 2022 at The First Affiliated Hospital of Nanjing Medical University were reviewed. Patients below 18 years of age were excluded, as were consultation cases and those who solely underwent fine-needle aspiration or adrenal gland biopsies. Inclusion criteria mandated patients to have undergone adrenalectomy, with available paraffin-embedded blocks. The final study cohort comprised 78 patients.

### Data collection

Clinical records of the 78 selected patients were meticulously reviewed, and relevant data were systematically collected. This encompassed personal details (age, gender, contact information), clinical presentations, results of hormonal tests, diagnostic imaging data, details of therapeutic interventions, and postoperative monitoring. Long-term follow-up data, including outcomes and overall survival (OS), were obtained through a combination of recent medical records and telephone surveys.

### Morphological assessment

All specimens of adrenocortical neoplasms underwent a comprehensive morphological assessment. This evaluation followed established criteria, including the modified Weiss criteria and the Lin-Weiss-Bisceglia criteria for oncocytic variants. Parameters assessed included mitotic counts (> 5 per 50 high-power fields in hot spots), the presence of atypical mitotic figures, evidence of necrosis, capsular invasion, the proportion of clear cells (≤ 25%), and the identification of angioinvasion. Angioinvasion was defined as the gross or clinically detected invasion of large vessels or microscopic angioinvasion characterized by tumor cells invading vessel walls or intravascular tumor cells coexisting with platelet thrombi.

### Tissue microarray (TMA) construction

For TMA construction, a total of 78 specimens were carefully selected. Hematoxylin and eosin-stained sections were reviewed, and four circular regions were marked for each ACC specimen, acknowledging the heterogeneity within these tumors. A single circle was designated for each ACA specimen. Areas exhibiting necrosis were deliberately excluded from the selection. Each circle possessed a diameter of 1.5 mm. Columns of the same dimensions were extracted from donor paraffin blocks in the vicinity of these circles and systematically embedded into recipient paraffin blocks according to a predetermined order.

### Immunohistochemistry and histochemistry

TMA sections, each measuring 4 μm in thickness, underwent dewaxing in xylenes and subsequent rehydration using alcohol solutions. Antigen retrieval was performed through hyperbaric heating with citrate buffer. Endogenous peroxidase activity was blocked using a 3% hydrogen peroxide solution. Antibodies, optimally diluted, were applied and incubated overnight at 4℃. The color development process involved the use of 3,3’-Diaminobenzidine (DAB). Control experiments were conducted to refine antibody use, with particular focus on markers like IGF-2 (Abclonal, A2086), CYP11B1(Fuzhou Maixin Biotech, MX136), CYP11B1(Fuzhou Maixin Biotech, MX137) and PHH3(Proteintech, 4C7G2). In addition, Gomori’s Silver histochemistry was employed to assess the reticulin framework. Ki67 and reticulin staining were conducted in a whole slide, and other stainings were implemented in TMA sections.

### Statistical analysis

The statistical analysis employed SPSS Statistics 26. Age and tumor size were subjected to T tests and reported as mean, range, and median values. Gender, tumor location, symptoms, morphological features, immunohistochemical results, and histochemical results were analyzed using the chi-squared test (χ² test). Correlation analysis of two groups of data selects Pearson / Spearman / Kendallta analysis according to data types, and partial correlation analysis is used to control the influence of other variables. Logistic regression, Receiver Operating Characteristic Curve (ROC Curve) analysis, and the calculation of the Area Under Curve (AUC) were utilized to assess the diagnostic value of individual markers and to determine sensitivities and specificities. AUC values were categorized as < 0.7 (indicating low diagnostic value), 0.7 to 0.9 (relatively high diagnostic value), and > 0.9 (very high diagnostic value). Overall survival analysis was conducted using the Kaplan-Meier method to identify factors potentially impacting ACC patient outcomes, with statistical significance set at a P-value below 0.05.

## Results

### Clinical features

As detailed in Table 1, the patients included in this series were adults, with the mean age at the time of diagnosis ranging from 36 years to 84 years in ACC cases (mean: 55.3 years) and from 25 years to 68 years in ACA cases (mean: 54.4 years)(*P* = 0.718). Among the ACC patients, there were 12 male and 16 females, while the ACA group comprised 22 males and 28 females *(P* = 1.000).

The mean tumor size in ACC cases was 9.7 cm, with a range from 1.6 cm to 21.0 cm, which was significantly larger than that in ACA cases (the mean tumor size was 2.7 cm, with a range from 0.7 cm to 7.0 cm) (*P*<0.001). A cutoff value of 4.3 cm for tumor size indicated that when a tumor exceeded this size, malignancy should be considered in this series.

12 ACC and 10 ACA patients were admitted to the hospital due to clinical symptoms, whereas the remaining individuals were diagnosed incidentally through imaging during routine physical examinations and were asymptomatic (*P* = 0.031), indicating a significant difference. Among the patients presenting with symptoms, 9 ACC patients reported experiencing pain, ranging from mild to intense. Additionally, 2 ACC patients exhibited endocrine symptoms related to adrenocortical hormones, such as hypertension and hypokalemia. 1 ACC patient reported the presence of a palpable mass. In contrast, all ACA patients with symptoms manifested endocrine-related symptoms. Preoperative plasma tests for adrenocortical hormones were conducted in 16 ACC cases and 30 ACA cases, and hormone levels for the remaining cases remained unknown. Elevated hormone levels existed in 5 ACC cases and 15 ACA cases(*P* = 0.222). Among them, 5 ACCs were cortisol-elevated and one of them accompanied aldosterone elevation. The 2 ACCs that manifested endodrine-related symptoms were both cortisol-elevated, and either of them was additionally aldoterone-elevated. 8 ACAs were cortisol-elevated (2 were symptomatic), 5 ACAs were aldosterone-elevated (all symptomatic) and 3 were simultaneously cortisol- and aldoterone-elevated (all symptomatic). Elevated hormone levels statistcally correlated with endodrine-related symptoms (*P*<0.001).

Regarding tumor location, 14 ACCs was found in the left retroperitoneum, 13 in the right retroperitoneum, and 1 involved both sides. Among the ACA cases, 24 were located in the left retroperitoneum, and 26 were in the right retroperitoneum (*P* = 0.384).

At the time of data collection, 13(46%) ACC patients had passed away due to the disease, while 10(36%) were still alive. Unfortunately, 5(18%) ACC patients were lost to follow-up. The mean follow-up time for ACC patients was 52.9 months, ranging from 7 to 103 months. Overall survival within the group ranged from 0 to 92 months, with a median survival of 27 months.

### Morphology

**Fig. 1:**
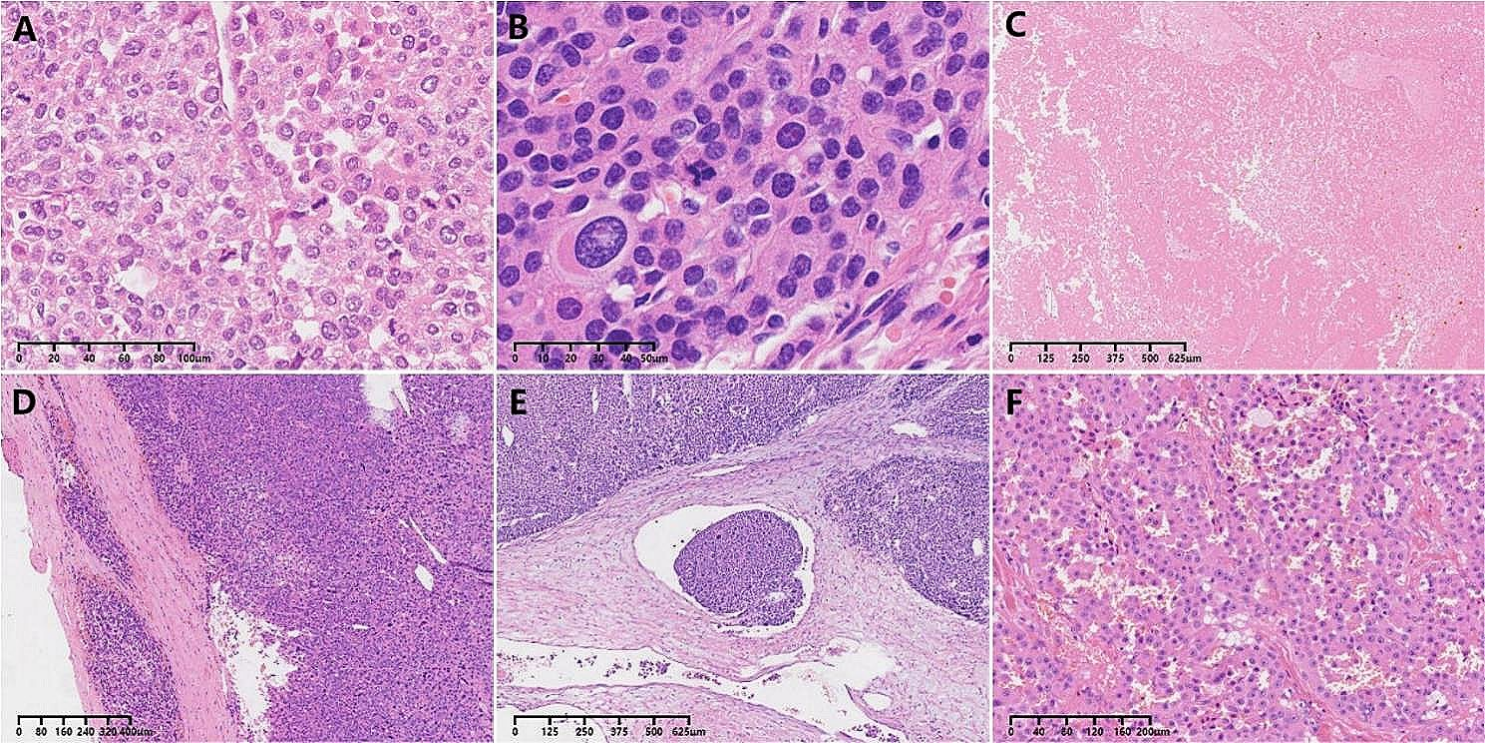
Morphology of ACCs. (**A**) The mitotic count of ACCs was commonly >5/50HPF; (**B**) Atypical mitosis; (**C**) Extensive tumorous necrosis of ACC; (**D**) Capsular invasion; (**E**)Angioinvasion; (**F**) A case of oncocytic ACC

The results of the morphological analysis are summarized in Table 1. In this series, 93%(26/28) of ACCs exhibited mitotic counts exceeding 5/ 50 HPF, whereas no mitotic figures were observed in 50 ACAs (*P*<0.001) (Fig. 1A). Atypical mitosis was identified in 39%(11/28) of ACCs, while ACAs showed no signs of atypical mitosis (*P*<0.001)(Fig. 1B). Necrosis was present in 79%(22/28) of ACCs but was not observed in ACAs (*P*<0.001)(Fig. 1C). In 89%(25/28) of ACCs, tumor cells were seen invading the capsule, with 2 of them extending through the capsule and involving extracapsular fat. In contrast, the capsules of all ACAs remained intact, with no tumor cell extension into or through the capsule(*P*<0.001)(Fig. 1D). Clear cells accounting for ≤ 25% of the total tumor composition were observed in 57%(16/28) of ACCs and 6%(3/50) of ACAs(*P*<0.001). Angioinvasion was noted in 32%(9/28) of ACCs, while it was absent in all ACAs. Among the 9 ACCs with angioinvasion, 1 exhibited gross large vessel invasion, while the others showed microscopic intravascular tumor thrombus(Fig. 1E).

**Table 1:** Clinical features and Morphology of Adrenocortical Carcinomas and Adrenaocortical Adenomas

	Adrenocortical Carcinomas (*n* = 28) *n*(%)	Adrenaocortical Adenomas (*n* = 50) *n*(%)	*P*
Mean age at presentation(y)	55.3(36.0–84.0)	54.4(25.0–68.0)	0.718
	(median: 58.0)	(median: 57.0)	
Gender			
Male	12(43)	22(44)	
Female	16(57)	28(56)	1.000
Mean tumor size(cm)	9.7(1.6–21.0)	2.7(0.7-7.0)	<0.001
	(median: 9.5)	(median: 2.85)	
Symptoms			
Asymptomatic	16(57)	40(80)	
Symptomatic	12(43)	10(20)	0.031
Pain	9(32)	0(0)	
Endocrine	2(7)	10(20)	
Palpable mass	1(4)	0(0)	
Hormone level			
Elevated (cor: cortisol; ald: aldosterone)	5(18) (4 caes: cor; 1case: cor + ald)	15(30) (8 cases: cor; 5cases: ald; 3 cases: cor + ald)	
Normal	11(39)	15(30)	0.222
Tumor site			
Left	14(50)	24(48)	
Right	13(47)	26(52)	
Bilateral	1(3)	0(0)	0.384
Mitotic count>5/50HPFs	26(93)	0(0)	<0.001
Atypical Mitosis	11(39)	0(0)	<0.001
Necrosis	22(79)	0(0)	<0.001
Clear cells ≤ 25%	16(57)	3(6)	<0.001
Capsular invasion	25(89)	0(0)	<0.001
Angioinvasion	9(32)	0(0)	<0.001
Mean Modified Weiss score	5.07(2–7)	0.06(0–2)	<0.001

In this series, the mean modified Weiss score for ACCs (5.07, ranging from 2 to 7) was significantly higher than that for ACAs (0.06, ranging from 0 to 2). Two cases did not meet the modified Weiss criteria for malignancy, as they only displayed necrosis and capsular invasion. Notably, these 2 cases also exhibited nuclear atypia, which was indicative of a high Fuhrman nuclear grade. However, when applying the Weiss scoring system, these 2 cases were diagnosed as ACCs. No sarcomatoid or myxoid ACC cases were identified in this series. 7(25%) specimens exhibited a predominant and pure oncocytic cytomorphology (comprising over 90% of the total), which were classified as oncocytic ACCs according to the Lin-Weiss-Bisceglia criteria(Fig. 1F). The other 21 ACCs were of the conventional type.

### Features of immunohistochemistry and histochemistry

The results and AUCs of immunohistochemistry and histochemistry are summarized in Tables 2 and 3. During the optimization of IGF-2 antibodies, a high dilution rate was necessary to reduce the basal normal reactivity, which was observed in almost all tumor cells. Satisfactory staining in the specimens was achieved with dilutions of 1/4000 for IGF-2. Initially, human tonsillitis tissue was used as a positive control for PHH3, and mitosis was better labeled at dilutions of 1/4000 and 1/6000. It was observed that cells in the interphase of division were slightly stained in the nucleus, which did not affect the distinction of strongly labeled mitotic cells. Nevertheless, tonsillitis tissue displayed predominant mitotic staining in the middle area, but the edge area showed poor contrast due to the strong positive staining of interphase cells, making it challenging to distinguish from mitosis. Subsequently, Burkitt lymphoma, characterized by a high mitotic rate, was included in the experiment and showed the same staining pattern as tonsillitis. However, when PHH3 was used in ACCs, interphase cells were slightly positive, while all mitotic cells were completely unstained, which differed from tonsillitis and Burkitt lymphoma. A definitive explanation for this phenomenon was not reached.

**Fig. 2:**
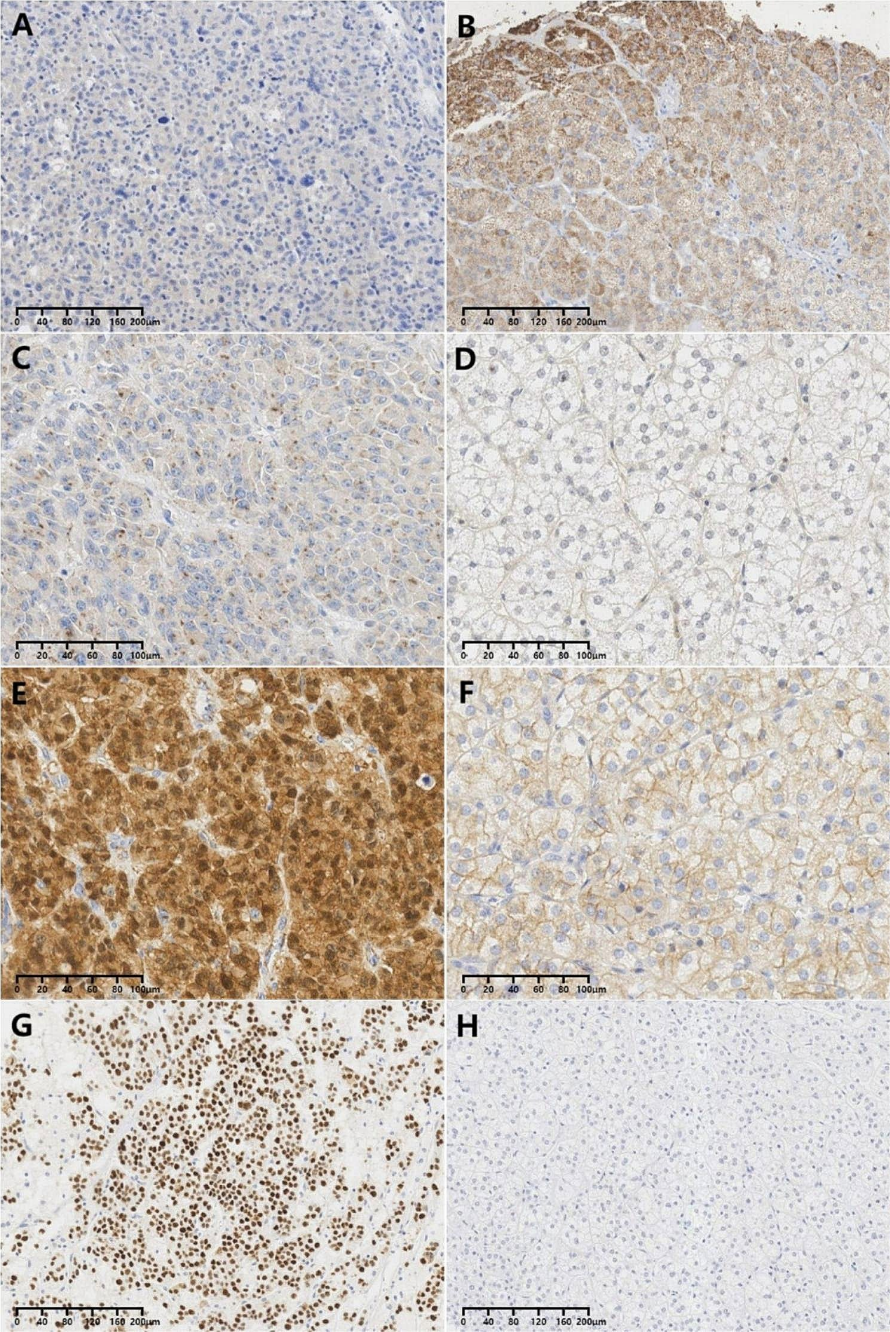
Immunohistochemistry. CYP11B1 showed cytoplasmic negativity more frequently in ACCs (**A**) than ACAs (**B**); IGF-2 showed juxtanuclear granular positivity specific to ACCs (**C**); ACAs displayed global negativity (**D**). Nuclear reactivity of β-catenin was specific to ACCs (**E**); ACAs were menbranous and/or cytoplasmic positivity (**F**); P53 overexpression could be identified in ACCs (**G**); ACAs showed weak or negative P53 expression (**H**)

15(54%) ACCs and 38(76%) ACAs showed CYP11B1 cytoplasmic staining, (*P* = 0.042)(Fig. 2A and B). CYP11B1 positivity had no correlation with endocrine-related symptoms. Due to the lack of preoperative hormone data and its correlation with endocrine-related symptoms, we use endocrine-related symptoms to evaluate the relationship between hormone and CYP11B1 expression. When controling the influence of endocrine-related symptoms, CYP11B1 expression was still significantly different between ACCs and ACAs (*P* = 0.028). The AUC of CYP11B1 was 0.612 and sensitivity(46.4%) and specitivity(76.0%) were relatively low. No statistical difference between ACCs and ACAs in CYP11B2 expression (*P* = 0.796). CYP11B2 cytoplasmic staining correlated with endocrine-related symptoms (*P*<0.001).

IGF-2 expression was observed in 13(46%) ACCs, whereas only 10(20%) ACAs displayed focal and weak cytoplasmic positivity(*P* = 0.014) (Fig. 2C and D). Among these ACCs, all showed juxtanuclear granular expression, with 6 displaying additional cytoplasmic staining. However, no ACAs exhibited this juxtanuclear granular pattern. Juxtanuclear granular expression of IGF-2 was considered a distinctive diagnostic marker for carcinomas in this study. IGF-2 exhibited a specificity of 100%, along with a sensitivity of 46.4%, and an AUC of 0.732. Overall, IGF-2 was valuable in diagnosing ACCs with high specificity but low sensitivity.

β-catenin exhibited different staining patterns in ACCs and ACAs. ACCs showed cell membranous, cytoplasmic, and nuclear staining, either singly or simultaneously, whereas ACAs displayed membranous and/or cytoplasmic expression, except for nuclear staining (Fig. 2E and F). Among ACCs, 9(32%) specimens showed nuclear β-catenin staining in at least nests of cells, which significantly differed from ACAs(*P*<0.001). β-catenin exhibited a specificity of 100% and a sensitivity of 32.1%, with an AUC of 0.661. These results indicated that β-catenin was a diagnostic marker with low effectiveness for the identification of carcinomas.

ACCs displayed a staining pattern of global loss, wild-type expression, and overexpression for P53. ACAs universally exhibited low expression, with only weak nuclear positivity in a few cells or were globally negative. This made it difficult to differentiate ACAs from ACCs with wild-type expression and global loss (Fig. 2G and H). “Overexpression” was used as a parameter for P53 expression in this study. P53 overexpression was observed in only 4 ACCs (14%), which significantly differed from ACAs*(P* = 0.027). P53 overexpression had a specificity of 100% and a sensitivity of only 14.3%. Its AUC value was 0.571, indicating that P53 overexpression was helpful but not highly efficient for the diagnosis of carcinomas in this series.

**Fig. 3:**
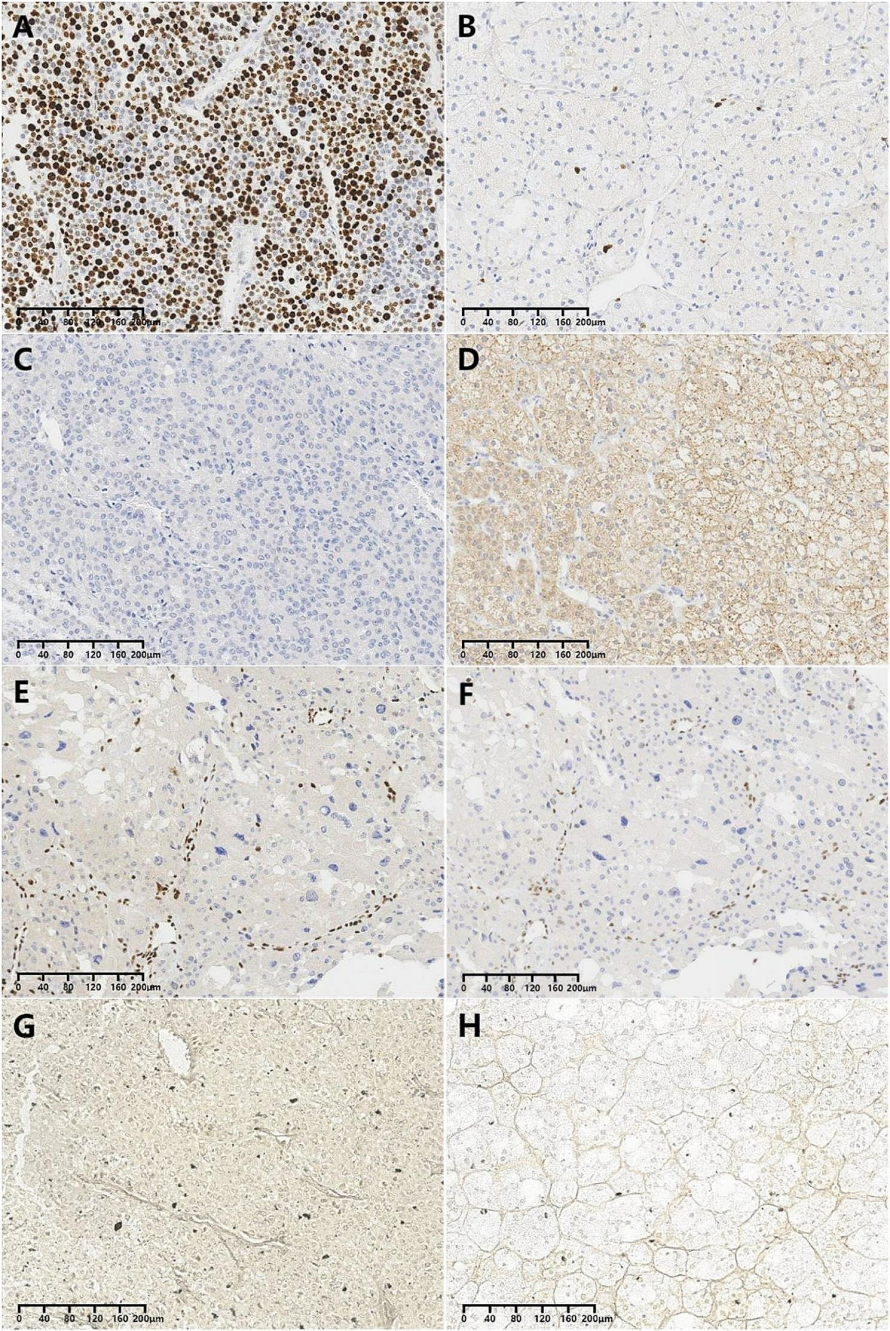
Immunohistochemistry and histochemstry. Ki67 labeling index of ACCs (**A**) was greater than that of ACAs (**B**); Some ACCs were MelanA-negative (**C**); ACAs showed MelanA cytoplasmic positivity (**D**); An ACC case showed MSH2 (**E**) and MSH6 (**F**) negativity, considered to be MMR-deficient. Evidently altered reticulin framework was commonly noted in ACCs (**G**); Reticulin framework of all ACAs kept intact (**H**)

22(79%) ACCs had Ki-67 labeling indices exceeding 5%, whereas all ACAs had a Ki-67 index of less than 5%(*P*<0.001)(Fig. 3A and B). The sensitivity and specificity of Ki67 labeling index were 85.7% and 100%, respectively, with an AUC of 0.909, suggesting that the Ki-67 index was a useful and highly effective diagnostic indicator of ACCs.

Regarding markers of adrenal cortex origin, Calretinin exhibited cytoplasmic positivity in 17(61%) ACCs and 28(56%) ACAs(*P* = 0.686). Alpha-Inhibin was cytoplasmically positive in 25(89%) ACCs and 37(74%) ACAs(*P* = 0.109). SF-1, considered the best marker for adrenal cortex origin, showed strong nuclear positivity in all ACCs and ACAs, making it statistically not applicable as a differentiating factor. MelanA displayed significantly different cytoplasmic expression in 23(82%) ACCs compared to all ACAs(*P* = 0.009). Additionally, the positivity of 23 ACCs was generally weaker than that of ACAs (Fig. 3C and D). The sensitivity, specificity, and AUC of MelanA were 17.9%, 100%, and 0.589, indicating that its diagnostic value was low.

All ACAs were found to be MMR-proficient in this series, with the four MMR proteins (MLH1, MSH2, MSH6, and PMS2) displaying nuclear expression. However, 2 ACCs were MMR-deficient; 1 showed negativity in MSH2 and MSH6, and the other exhibited MSH6-negativity (Fig. 3E and F)(*P* = 0.243). All ACCs and ACAs were EPCAM-negative.

24(86%) ACCs and no ACAs displayed evident alterations in the reticulin framework, which showed statistical significance (*P*<0.001)(Fig. 3G and H). The AUC of this feature was 0.929, with high sensitivity (85.7%) and specificity (100%), indicating that evident alteration of the reticulin framework was a valuable indicator of carcinomas.

In summary, the recommended methods for ACC identification in this study were immunohistochemistry of Ki-67 and Gomori’s silver histochemistry.

**Table 2:** Features of Immunohistochemistry and Histochemistry of **Adrenocortical Carcinomas and Adrenaocortical Adenomas**

	Adrenocortical Carcinomas (*n* = 28) *n*(%)	Adrenaocortical Adenomas (*n* = 50) *n*(%)	*P*
CYP11B1			
positive	15(54)	38(76)	<0.042
CYP11B2			
positive	6(21)	12(24)	
IGF-2			
Positive	13(46)	10(20)	0.014
Juxtanuclear granular pattern	13(46)	0(0)	<0.001
β-catenin			
Nuclear expression	9(32)	0(0)	<0.001
P53			
Overexpression(>5%)	4(14)	0(0)	0.027
Ki-67			
Mean labeling index(%)	27(2–80) (median: 20)	2.5(2–3) (median: 3)	<0.001
>5%	22(79)	0(0)	<0.001
Calretinin			
Positive	17(61)	28(56)	0.686
Alpha-Inhibin			
Positive	25(89)	37(74)	0.109
SF-1			
Positive	28(100)	50(100)	N/A
MelanA			
Positive	23(82)	50(100)	0.009
MMR-deficient	2(7)	0(0)	0.243
Recticulin framework			
Evidently altered	24(86)	0(0)	<0.001

**Table 3:** AUC Values of ROC Curve of Immunohistochemistry and Histochemistry

	AUC	Cut-off	Sensitivity	Specificity	*P*
Ki67	0.909	0.857	0.857	1.000	<0.001
P53	0.571	0.143	0.143	1.000	0.298
MelanA	0.589	0.179	0.179	1.000	0.193
IGF2(Juxtnuclear granular)	0.732	0.464	0.464	1.000	0.001
β-catenin	0.661	0.321	0.321	1.000	0.019
CYP11B1	0.612	0.224	0.464	0.760	0.102
Reticulin framework	0.929	0.857	0.857	1.000	<0.001

### Prognostic value of clinical and pathological features, immunohistochemical markers and Gomori’s silver histochemistry

**Fig. 4:**
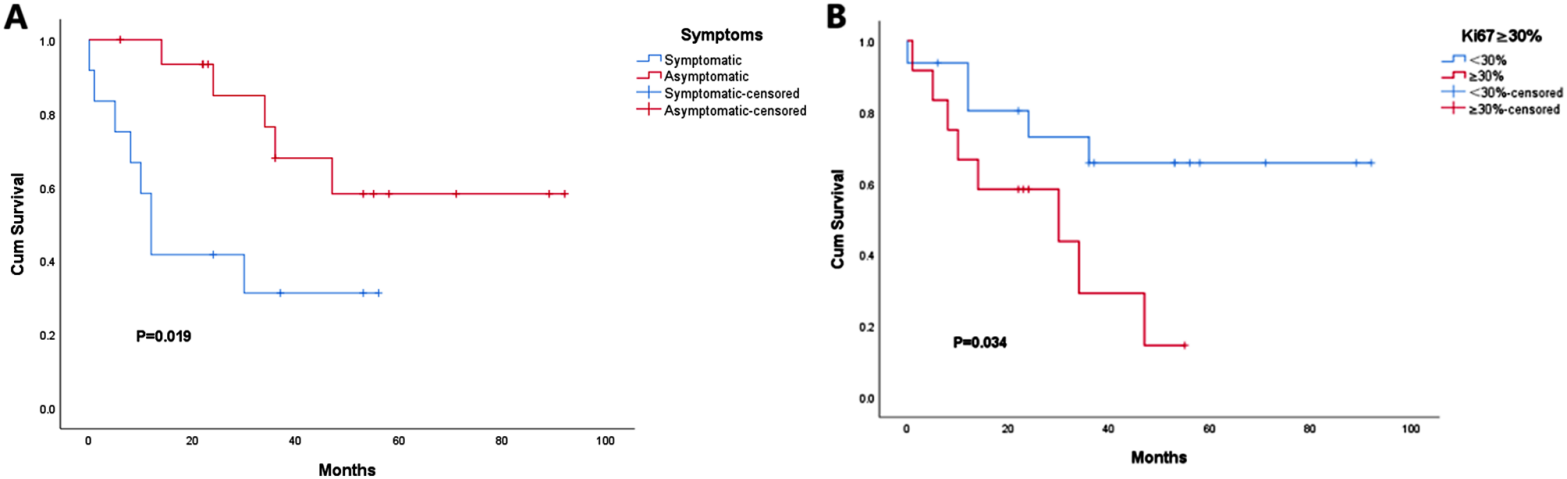
Asymptomatic patients diagnosed with ACC generally have longer OS than symptomatic ones, indicating that the presence of clinical symptoms is a predictive feature of outcomes (**A**); Increasing Ki-67 labeling index correlates worse outcomes when a cutoff of 30% was applied(**B**)

Survival analysis was conducted to assess the prognostic value of various features of ACCs on overall survival (OS). Among the clinical features, it was found that symptoms at the time of hospitalization were significantly correlated with overall survival(*P* = 0.019). Asymptomatic patients had a better survival outcome compared to those with symptoms (Fig. 4A). However, age, gender, tumor size, tumor location, and modified Weiss scores did not show any significant correlation with overall survival.

Similarly, among the morphological features analyzed, including mitotic count, atypical mitosis, necrosis, capsular invasion, percentages of clear cells, and angioinvasion, none displayed a significant correlation with OS in this series.

Regarding immunohistochemical markers and reticular histochemical expression, only the Ki-67 labeling index, using a cutoff of 30%, showed statistical significance in relation to OS. This indicated that ACCs with a Ki-67 labeling index of ≥ 30% had a worse prognosis compared to those with a lower index (*P* = 0.034)(Fig. 4B). However, other markers did not demonstrate a significant prognostic correlation.

### Comparison of features between subtypes

Among the 28 ACCs analyzed in this study, they were further classified into 21 conventional ACCs and 7 oncocytic ACCs based on modified Weiss criteria and Lin-Weiss-Bisceglia criteria. An analysis was performed to assess various features, and the results revealed that the frequency of necrosis was significantly higher in conventional ACCs compared to oncocytic ACCs, with 19 cases vs. 3 cases, respectively (*P* = 0.033). However, all other analyzed features did not show a statistically significant difference between these two subtypes of ACCs.

## Discussion

In this study, the results indicated that ACCs tend to exhibit larger tumor sizes, in line with previous findings reporting that ACAs are typically smaller than 5 cm, while ACCs are notably larger [1]. ACCs also tend to present clinical symptoms, which may be associated with worse outcomes [13]. When clinical symptoms, especially pain, coincide with the presence of an adrenocortical mass, malignancy should be considered. Asymptomatic patients generally have a better prognosis, likely because asymptomatic cases are often in the early stages of cancer, and early adrenalectomy may extend survival.

All the morphological features we mentioned proved valuable for diagnosing ACCs in our study. Except for the percentage of clear cells, the other features were specific to ACCs. However, existing diagnostic criteria typically require the presence of multiple malignancy-related features for an ACC diagnosis, as inter-observer variability can lead to inconsistencies in identifying certain features, such as mitotic count per 50 HPF, atypical mitosis, percentage of clear cells, capsular and vascular invasion. Relying solely on a single malignancy-related feature poses a high risk of misdiagnosis [3–5]. Therefore, while each morphological feature, except the percentage of clear cells, is specific for identifying malignancy, adopting a one-feature strategy is not advisable. Furthermore, some moderate or low-grade ACCs may not meet established diagnostic criteria, similar to the two cases in our study that did not satisfy the modified Weiss criteria. In our quest for a more intuitive and effective method for ACC diagnosis, we conducted experiments to determine whether immunohistochemistry and Gomori’s Silver histochemistry, based on tissue microarrays, could differentiate adenomas from carcinomas.

Regarding the prognostic analysis, although the 2022 WHO classification highlighted the prognostic impact of angioinvasion, our study failed to support this proposal [3]. Similarly, other morphological features did not prove predictive of outcomes.

Among all immunohistochemical markers and histochemical staining of the reticulin framework, the most valuable diagnostic feature in our study was the obvious alteration of the reticulin framework, followed by Ki-67 labeling index greater than 5%. ACCs with a Ki-67 labeling index of 30% or more were associated with worse outcomes.

The recognition of alterations in the reticulin framework has undergone a developmental process. The “Reticulin Algorithm (RA),” initially proposed by Volante et al. [14], defines the malignancy of adrenocortical neoplasms based on the disruption of the reticulin framework along with at least one of the following parameters: a mitotic rate greater than 5/50HPF, tumor necrosis, or angioinvasion. Disruption of the reticulin framework is characterized by the loss of continuity of reticular fibers or the basal membrane network in one HPF, existing in at least one-third of lesions. Volante et al. found that RA had 100% sensitivity and specificity for identifying malignancy based on the Weiss system, while disruption of the reticulin framework alone was 100% sensitive and 96% specific [14]. Subsequently, Duregon et al. [15] conducted a multicentric analysis in 245 adrenocortical cases, applying both the Weiss scoring system and RA. They introduced the concepts of “quantitative” and “qualitative” alteration of the reticulin framework. Quantitative alteration was defined as proposed by Volante et al., while qualitative alteration was characterized by an intact reticulin framework with fibers varying irregularly in thickness, surrounding single cells or small groups of cells. Their study included both quantitative and qualitative alterations. Interestingly, 201 cases exhibited alterations in the reticulin framework, yet ultimately, only 178 cases were diagnosed as ACC by RA (184 ACCs by Weiss criteria, and 6 ACCs were reclassified as ACAs by RA). The diagnostic consistency of the two method was good. Duregon’s study confirmed the viability of RA but also raised questions about the definition of alterations in the reticulin framework. Subsequently, Mete et al. [2] emphasized that quantitative alteration of the reticulin framework was more valuable in distinguishing ACCs from ACAs. Angelousi et al. [16] demonstrated the feasibility of the quantitative alteration method in ACCs. In this study, we discussed the application of reticulin staining alone in the identification of carcinomas and found that the results supported the utility of obvious alteration of the reticulin framework (quantitative alteration) while diminishing the diagnostic significance of the pericellular pattern (qualitative alteration), consistent with previous studies [2, 14]. Importantly, quantitative alteration is more accessible and repeatable in the practice of reticular histochemistry, as the reticulin framework is predominantly disrupted and easy to recognize. In contrast, the observation of qualitative alteration is subtle and challenging to determine, which may lead to inter-observer disagreements and even mistaken judgments. Additionally, qualitative alteration is also present in ACAs, reducing the specificity of reticulin histochemistry and increasing the risk of misdiagnosis [2, 3]. In conclusion, the utility of reticulin histochemistry based on quantitative alteration is highly recommended for distinguishing ACCs from ACAs.

CYP11B1 and CYP11B2 (cytochrome P450 family 11 subfamily B member 1 and 2), known as a functional marker, has been used to evaluate the functional status of adrenocortical pathology in previous studies but has rarely been discussed in the context of recognizing malignancy in adrenocortical neoplasms [3, 8, 17]. According to urine steroid metabolomics, most ACCs exhibit a pattern of immature, early-stage steroidogenesis, and CYP11B1, as an enzyme in the steroid pathway responsible for converting cortisol, is relatively deficient in ACCs. This implies that ACCs may have low levels of CYP11B1 proteins [18, 19]. Uchida et al. [20] reported a case of ACC causing mild primary aldosteronism and subclinical Cushing’s syndrome, in which the immunohistochemistry of CYP11B1 showed loss of expression in most areas. Yang et al. [21] tested the immunoreactivity of CYP11B1 in a series of 9 ACCs and 190 ACAs with different functional statuses and found the lowest immunoreactivity in ACCs, with an AUC of 0.976, indicating that CYP11B1 has the potential for diagnosing ACC. Pereira et al. [22] conducted immunohistochemistry in a series of 14 ACCs and 26 ACAs and found that ACCs had decreased expression of CYP11B1 compared to ACAs. They were the first to announce that CYP11B1 had discriminative power in differentiating carcinomas from adenomas. Our results showed statistical difference between two groups but the AUC value was relatively low, suggesting that CYP11B1 had potential for distinguishing carcinomas from adenomas. Additionally, Qing Chen et al. [23] reported the identification of a CYP11B1 signature in ACCs and its prognostic value. However, our study showed no prognostic significance of CYP11B1. CYP11B2 did not manifested diagnostic potential for ACC, but it could be used to evaluate the function status and located the secreting area of aldosterone-secreting adrenocortical neoplasms.

Ki-67 is a classical marker that reflects the activity of cell proliferation and is widely used in various tumors. ACC, as a proliferation-driven malignancy, typically displays a Ki-67 labeling index greater than 5%, which significantly exceeds that of ACA [3, 5, 23]. Notably, ACCs exhibit proliferative heterogeneity, requiring the accurate identification of hot spots to precisely assess the Ki-67 labeling index. Our study supports the diagnostic value of the Ki-67 labeling index in assessing malignancy in adrenocortical tumors, with a specificity of 100%. Another critical use of Ki-67 in ACCs is risk stratification, with two schemes proposing three-tiered cut-offs (less than 10%, 10–19%, and 20% or more/less than 20%, 20–50%, and more than 50%) to categorize three prognostic groups [9, 24]. The higher the Ki-67 labeling index, the worse the prognosis for ACC patients. However, neither of these two schemes achieved their intended purpose in our study. Our results emphasized the prognostic role of the Ki-67 labeling index in ACC, underscoring a cut-off value of 30% for prognosis. Other levels of Ki-67 labeling index did not reveal any prognostic relevance.

PHH3, another proliferative marker specific to mitosis, is increasingly being used in various tumors to evaluate mitosis. PHH3 has advantages in assessing mitotic count compared to visual observation [9, 25, 26]. PHH3 can distinguish mitotic figures from apoptotic bodies, which are sometimes difficult to identify through microscopic observation of H&E slides, thereby improving the accuracy of mitosis identification [23, 24]. Additionally, the predominant staining of PHH3 by immunohistochemistry is time-saving and reproducible among observers. Few studies have performed PHH3 immunohistochemistry in ACCs, and its utility has not been fully validated [9].

IGF-2 (insulin-like growth factor 2) is known as a growth factor involved in cell proliferation and differentiation, stimulating steroidogenesis in the IGF pathway in the normal adult cortex. IGF-2, as detected by immunohistochemistry, has been considered the most established marker for diagnosing ACCs [3, 7]. Variable cytoplasmic IGF-2 staining was initially reported between ACCs and ACAs and was considered a useful feature for diagnosing carcinomas [27, 28]. Subsequently, another different juxtanuclear granular/Golgi staining pattern of IGF-2 was described, which was only observed in ACCs [2, 29]. Our study observed both cytoplasmic and juxtanuclear granular staining, with the latter being specific to ACCs, confirming the diagnostic value of IGF-2. It should be noted that the coexistence of different staining patterns in this series suggests that juxtanuclear granular reactivity requires strict identification to eliminate interference from cytoplasmic expression.

MelanA has been used as a biomarker for labeling adrenocortical origin. In this series, staining frequency and intensity differed between ACCs and ACAs [3, 7]. Whether this discrepancy is due to sampling error or represents a reliable result requires further research. Other markers of origin (SF-1, Calretinin, and alpha-inhibin) failed to distinguish ACCs from ACAs.

Gain-of-function mutations in β-catenin are found in approximately 25% of both benign and malignant sporadic adrenocortical neoplasms [30]. Diffuse nuclear β-catenin expression was previously confirmed as a feature of adrenocortical malignancy and was more frequent in tumors with adverse outcomes [2]. Our results further validated the adoption of β-catenin immunohistochemistry for assessing malignancy. However, due to the low frequency of nuclear expression, β-catenin did not demonstrate high diagnostic value, and no correlation was found between nuclear β-catenin expression and overall survival.

TP53 mutations are found in 20% of ACCs, resulting in functional disruption of the p53 protein [3]. P53 has been considered a marker of malignancy in adrenocortical neoplasms when showing abnormal p53 expression (overexpression or global loss) [3]. However, only 14% of carcinomas in this series displayed distinguishable overexpression from adenomas. Like previous studies, all ACAs showed low levels of p53 expression (less than 5%) [2, 16]. Previous research suggested that abnormal p53 expression might be identified in a subset of ACCs reflecting poor-prognostic molecular clusters and claimed that p53 overexpression was an independent predictor of a worse prognosis in metastatic ACCs [3, 28, 31]. However, our study did not confirm the prognostic significance of p53 overexpression.

According to an analysis of whole-exome data, 4.3% of ACCs revealed microsatellite instability (MSI), resulting from MMR impairment [11]. MSI is associated with susceptibility to immune-enhancing therapies and a better prognosis [32]. Immunohistochemistry of MMR proteins is increasingly used for primary MSI testing [3]. In this study, two MMR-deficient cases were malignant. Although this finding was not statistically significant, MMR deficiency could be a feature of ACC, warranting further investigation. As for EPCAM, deletions of the 3’ end of this gene could lead to inactivation of the promoter of the MSH2 gene downstream, resulting in complete negativity in all cases, as well as in normal adrenocortical tissue in this study, suggesting that poor expression of EPCAM might be a characteristic of adrenocortical origin [33].

## Conclusion

This study underscores the diagnostic significance of conspicuous alterations in the reticulin framework and a high Ki-67 labeling index for ACCs. Markers such as CYP11B1, IGF2, P53, β-catenin and MelanA also contribute to the diagnosis of ACCs. Patients presenting with tumor-related symptoms or a high Ki-67 labeling index (≥ 30%) exhibited a considerably reduced overall survival. Conventional ACCs displayed a higher frequency of necrosis compared to oncocytic variants. Although several multiparametric diagnostic criteria have been proposed and routinely applied in ACCs [3], they are mostly based on morphology, which is time-consuming and less reproducible. Our study encourges the use of ancillary markers such as Ki67 and reticulin histochemistry in the workup of evaluations of adrenocortical neoplasms, which are efficient, reproducible and easy-to-apply.

### Electronic supplementary material

Below is the link to the electronic supplementary material.

Supplementary Material 1

Supplementary Material 2

Supplementary Material 3

## Data Availability

Data is provided within the manuscript or supplementary information files.
